# Nutritional Dimensions of Sports Tourism: Runners’ Encounters with Polish Local Food Cultures

**DOI:** 10.3390/nu17162601

**Published:** 2025-08-10

**Authors:** Mateusz Rozmiarek

**Affiliations:** Department of Sports Tourism, Faculty of Physical Culture Sciences, Poznan University of Physical Education, 61-871 Poznan, Poland; rozmiarek@awf.poznan.pl

**Keywords:** sports tourism, sport, tourism, running, local cuisine, cultural exploration, nutrition, half marathon, Poznan, Poland

## Abstract

**Background/Objectives:** Although nutrition is widely recognized as a key factor in post-event recovery in sports, little attention has been given to how its cultural and social dimensions—embodied in local cuisine—intersect with the needs of traveling athletes, for whom food often also serves as a medium of cultural immersion and sensory exploration. Poland, with its rich regional culinary traditions and numerous international running events, offers a compelling context in which to explore these interactions. This study aims to understand the role of local cuisine in the experiences of foreign runners participating in the Poznan Half Marathon 2025, with particular attention on cultural engagement, tourist motivations, and post-exercise recovery processes. **Methods:** This study was based on a qualitative approach, utilizing semi-structured in-depth interviews conducted with 12 international runners from the United Kingdom, Germany, and Ukraine. The participants possessed a minimum of two years’ experience in traveling for sports. **Results:** The findings identified three main areas of the significance of food: (1) food as an element of cultural exploration, (2) local cuisine as a motivator or barrier when choosing a race, (3) food as a symbolic reward and structured recovery practice supporting nutritional and psychological processes. Approaches varied by nationality—British participants preferred spontaneous taste discovery, Ukrainians valued culinary comfort similar to home, and Germans planned their culinary experiences with greater awareness. **Conclusions:** Local cuisine plays a multifaceted role in international running events, serving not only nutritional needs but also emotional and cultural functions that shape the overall participant experience. Both event organizers and local restaurants should consider offering diverse and culturally sensitive food options to enhance recovery, satisfaction, and the appeal of sports tourism destinations.

## 1. Introduction

Over the past two decades, sports tourism has become one of the most dynamically developing segments of global mobility [1,2,3]. An increasing number of people travel not only to passively attend sporting events as spectators, but also as active participants, taking part in triathlons, cycling competitions, or running events [4,5,6,7]. Events such as marathons and half marathons attract not only local runners but also numerous international participants who combine their sporting engagement with a desire to discover new places. This form of mobility constitutes a specific case of experiential tourism [8], where physical activity intertwines with urban exploration, interpersonal encounters, and engagement with local cultures [9].

One of the often underestimated yet significant aspects of sports tourism is the experience of local cuisine, which serves both nutritional and cultural functions. Polish cuisine is characterized by its hearty and diverse regional dishes, deeply rooted in centuries-old traditions that reflect the country’s agrarian past [10]. Typical ingredients include various types of meats, potatoes, cabbage, and seasonal vegetables, often prepared using techniques like pickling, fermentation, and slow cooking. Traditional dishes such as pierogi (dumplings), bigos (hunter’s stew), gołąbki (meat-stuffed cabbage), zupa grzybowa (wild mushroom soup), and żurek (sour rye soup) illustrate the rich culinary heritage that varies across regions [11]. Moreover, Poland’s local food culture emphasizes the importance of communal eating and seasonal produce, with many festivals and events celebrating traditional foods. For international visitors and sports tourists alike, encountering Polish cuisine offers not only nutritional sustenance but also an immersive cultural experience that connects them to the history and identity of the region [12].

For participants in sporting events outside their home country, being in a new place involves not only interaction with the city’s topography or its sports infrastructure but also—often intuitively—with its culinary heritage. For many, local cuisine acts as a carrier of cultural and regional identity [13,14], and it often constitutes the first point of contact between the tourist and distinctive sensory characteristics expressed through taste, smell, and texture. However, it may also present challenges due to unfamiliar flavors or dietary incompatibilities [15]. Contemporary research in culinary tourism and the anthropology of food suggests that the experience of meals can play a mediating role: connecting the tourist to a place through the senses, rituals, and narratives accompanying food [16,17,18]. In the case of athletes, the exploration of local flavors is often part of the broader spectrum of their stay—even if it is not the primary motivation for travel, it can play a key role in shaping memories and perceptions of hospitality and the overall assessment of the participation experience.

Nutrition in the immediate post-race phase holds special significance, contributing not only to athletes’ sense of reward but also to their physiological regeneration. The process of rebuilding the body after intense physical exertion relies on the timely and conscious intake of nutrients—carbohydrates, proteins, electrolytes, and fluids—whose replenishment supports metabolic processes and reduces the risk of overstrain [19]. In the sports nutrition literature, the concept of nutritional recovery emphasizes the importance of both the quality and timing of meals for the effectiveness of physiological regeneration [20,21]. However—as is particularly important from an anthropological and cultural perspective—nutrition does not occur in a vacuum, but is embedded in specific cultural practices and social contexts [22]. Foreign athletes are confronted with the local gastronomic offering, which may differ from their accustomed norms in terms of ingredients, presentation, taste, or symbolism. In such cases, the meal ceases to be merely a biological necessity—it becomes part of a culinary adventure, a moment of entry into local reality. For many athletes, the eating experience lies somewhere on a continuum between a return to familiar products (e.g., international food chains) and the exploration of local specialties, which may evoke both excitement and uncertainty. This creates a tension between the functional dimension of recovery and openness to cultural difference. Additionally, meals are often consumed in semi-public spaces—at the finish line, in rest zones, or in local restaurants—introducing a social and ritual dimension. Sharing food with fellow participants, discussing the course route, or celebrating one’s effort over a local dish—all of these moments shape a situation in which food becomes not only a physiological necessity but also a space of experience and exchange. In this context, recovery takes on both somatic and cultural dimensions, forming part of the complex experience of being a runner–tourist in an unfamiliar place.

Despite the growing interest in both sports tourism and tourists’ culinary experiences, existing research rarely attempts to integrate these two perspectives. The literature tends to maintain a division between the functional approach to nutrition in the context of physical exertion and the cultural–symbolic analysis of food as a social practice. There is a lack of in-depth studies showing how local cuisine becomes part of the sporting experience within the context of tourism, particularly among runners participating in events outside their home countries. While some studies have addressed the role of nutrition in maintaining the health and physical condition of sports volunteers [23,24], there is still a noticeable lack of research on how local cuisine contributes to the overall experience of international runners within the context of sports tourism. Moreover, Poland—as a host of numerous running events [25,26,27,28,29,30], and a country with a rich and regionally diverse culinary heritage [31,32,33]—constitutes an intriguing yet underexplored research field. This article addresses this gap by offering an approach that combines anthropological sensitivity, a tourism perspective, and an awareness of the physiological needs of runners—using the example of participants in the Poznan Half Marathon, Poland. Accordingly, the aim of this study is to understand the role of local cuisine in the experience of foreign runners participating in the half marathon in Poznan—with particular attention to cultural exploration, tourist motivations, and recovery-related aspects. This study is informed by established perspectives from sports tourism theory [4], food tourism research [34], and nutritional anthropology [35,36], which together help to frame the relationship between physical effort, cultural immersion, and food practices.

## 2. Materials and Methods

This study was based on a qualitative research design, with a primary focus on in-depth interviews (IDIs) [37]. As a methodological tool, IDIs are widely regarded as particularly effective for gaining rich insights into individual perspectives and for exploring a broad range of complex topics [38]. Conducting interviews in a one-on-one format—as opposed to group-based methods such as focus groups—enables more nuanced data collection and tends to foster a greater degree of openness and detail from participants, particularly when addressing sensitive or personal matters [39].

### 2.1. Sample Selection

A purposive sampling approach was used, guided by clearly defined inclusion criteria: (1) permanent residence outside Poland with travel undertaken specifically to participate in the Poznan Half Marathon 2025; (2) at least two years of experience in international travel for sports-related purposes. This form of sampling was selected to ensure the relevance of participants’ experiences to the study’s focus on international sports tourism and nutritional adaptation. To enhance access to eligible individuals and reach a broader range of information-rich cases, in accordance with Patton’s guidance [40], a snowball sampling strategy was additionally employed, wherein initial participants recommended other qualified runners.

The study involved twelve international runners, including eight men and four women. The participants were evenly distributed across three national groups: four from the United Kingdom, four from Germany, and four from Ukraine. The inclusion of participants reflected the actual demographic structure of foreign registrants for the Poznan Half Marathon 2025; these three countries accounted for the largest number of international runners at the event, which made their citizens the most accessible and information-rich population for recruitment. All individuals had a minimum of two years’ experience traveling abroad to participate in running events, thereby qualifying them as sports tourists, and held permanent residence in their respective home countries at the time of the study. The research concentrated on the role of local cuisine in the experiences of international runners participating in a running event, forming part of a broader investigation into the dynamics of the sports tourism experience, including strategies related to travel, nutrition, diet, and physical activity undertaken abroad. All interviewees were legally adults.

### 2.2. Justification for Sample Size

The sample size was established in accordance with qualitative research standards, which typically advocate for determining participant numbers based on the point of data saturation. However, in this study, saturation was not treated as a fixed numerical target but rather as a marker of conceptual completeness and thematic diversity, reflecting the point in data collection when additional interviews no longer produce new or significant themes that would enhance the understanding of the research topic, in line with current qualitative research conventions.

To ensure both depth and variation in the dataset, the participants were selected to reflect heterogeneity in terms of nationality, gender, and international travel experience. Additionally, individuals were not recruited from a single running club or national delegation, which minimized group-level bias and enhanced the contextual breadth of the data. The participants were selected to represent diverse approaches to sports travel and eating behavior, enabling the coverage of varied experiential dimensions. The iterative nature of data collection and analysis allowed for constant comparative analysis between interviews. After twelve interviews, the emerging thematic structure had become repetitive and no substantially new codes were identified. However, saturation was assessed not only through repetition but also through the variation across cases. The dataset was evaluated for multidimensionality to ensure that the themes reflected a wide range of perspectives rather than thematic redundancy. This involved comparing the richness and nuance of codes across participants and evaluating whether new interviews continued to expand the explanatory power of the coding framework. Numerically, these twelve interviews were also consistent with the recommendations found in the qualitative research literature [41,42].

### 2.3. Procedure

The research was carried out using semi-structured interviews, a format that allowed the participants to express their views freely through open-ended questions. All interviews were conducted in English during April 2025, with each session lasting approximately one hour. Recordings were made with the explicit consent of each participant. The entire study was conducted in accordance with established ethical standards for scientific research. The participants received comprehensive information regarding the study’s aims, procedures, and their right to voluntary participation. Informed consent was obtained from all individuals, both for participation and for the recording of the interviews. Confidentiality and anonymity were strictly upheld, and all data were reported in a manner that precluded the identification of any participant.

### 2.4. Data Analysis

#### 2.4.1. Thematic Coding Procedure

The data obtained through the interviews were analyzed using a structured four-phase thematic analysis process: (1) transcription of the audio recordings and initial immersion in the material; (2) categorization of participant responses and identification of recurring patterns; (3) organization of these categories into broader thematic groupings; (4) interpretation of findings and formulation of conclusions based on the emergent themes. Each phase was conducted in alignment with established qualitative research standards to ensure the trustworthiness and validity of the results.

The analysis was explicitly guided by Braun and Clarke’s [43] widely recognized framework for thematic analysis, which offers a systematic and transparent approach to identifying, analyzing, and reporting patterns within qualitative data. Employing this methodology enhanced both the credibility and replicability of the research process.

To deepen the analytical rigor, a multi-stage, iterative approach to thematic analysis was adopted. Following transcription, interview transcripts were reviewed multiple times to develop a thorough understanding of participant perspectives. Coding was performed in two distinct phases: an initial open coding phase to identify relevant themes, followed by axial coding to connect related codes and reveal core patterns. The analysis was supported by NVivo software (version 12), which facilitated the organization and management of the coding process, as well as the documentation of the entire analytic workflow, ensuring transparency and traceability consistent with established qualitative research practices. Although no visual coding trees were created, the development of themes followed a structured, multi-layered process. Codes were progressively abstracted into coherent categories and higher-level conceptual groupings.

Examples of initial codes included “local food as reward,” “culinary familiarity,” “trying Polish specialties,” “post-run indulgence,” and “pre-race meal concerns.” These codes were iteratively compared and organized into broader analytical groupings that reflected recurring patterns in how participants engaged with food while abroad. Such groupings included cultural exploration through food, the use of meals as recovery and reward, and the influence of culinary expectations on event choice. These categories were then examined for conceptual overlap, divergence, and explanatory value. Each theme was supported by multiple participant narratives and was cross-checked against raw data to ensure internal coherence, distinctiveness, and relevance to the research aims. Themes were finalized when they demonstrated conceptual clarity, strong internal consistency, and meaningful connection to the central research questions. The process involved iterative cycling between coded data and thematic groupings, guided by Braun and Clarke’s principles of coherence, distinctiveness, and resonance. Only those themes that were both recurrent and theoretically informative were retained in the final structure.

#### 2.4.2. Reflexive Approach and Analytical Rigor

All stages of the data analysis were conducted by the same researcher who also carried out the interviews. This approach promoted a uniform interview style and helped establish rapport with participants, encouraging more in-depth and candid responses. The semi-structured format, based on a standardized set of core questions, enabled comparability across interviews while preserving flexibility to explore individual experiences and perspectives more deeply. Although this decision limited opportunities for external verification, it ensured a high level of contextual immersion and interpretive continuity throughout the research process.

Given the exploratory and interpretive nature of the study, no inter-coder reliability checks or peer audits were performed. Instead, analytical rigor was maintained through reflexive thematic analysis, supported by a detailed reflective journal documenting coding decisions, emergent assumptions, and theoretical sensitivity. To mitigate confirmation bias and enhance reflexivity, moments of analytical uncertainty, divergent interpretations, and theoretical predispositions were explicitly recorded, creating an internal audit trail that served as an accountability mechanism during the inductive process [44].

Theoretical sensitivity developed iteratively through close engagement with both empirical data and the relevant scholarly literature, while remaining open to unanticipated meanings emerging from the material. The analysis was conducted with an awareness of the researcher’s positionality and the potential influence of prior assumptions, and the researcher actively worked to bracket these during coding and interpretation. Saturation was not assessed in isolation but monitored alongside the evolving richness and variation in themes across interviews. Themes were continuously cross-checked against initial codes and raw data to ensure consistency and credibility.

This approach aligns with current qualitative research standards that prioritize reflexivity and transparency over inter-rater consensus, especially in inductive designs that recognize the researcher as an interpretive instrument [44,45]. To further strengthen credibility and minimize unchecked subjectivity, previously coded material was periodically revisited to reassess early interpretations in light of new data, explicitly searching for contradictory or variant perspectives. This recursive self-check functioned as an internal dialogical mechanism of analytical control.

Throughout this study, the reflexive journal recorded all analytic decisions, emergent categories, and interpretive doubts, including moments of theoretical tension or confirmation bias. This practice allowed for the critical examination of emerging interpretations relative to prior assumptions and actively encouraged seeking disconfirming evidence. Transparency and critical reflection on the coding process and thematic interpretation were thus supported. The risk of premature closure was addressed through the critical comparison of early and late interviews, ensuring saturation was grounded in both repetition and representational richness. This approach aimed to achieve what the recent literature terms “meaning saturation” [46], defined as the point at which no new conceptual insights emerge from the data. This integrated and reflexive approach to data analysis ensured both methodological rigor and conceptual depth in interpreting the findings.

## 3. Results

The analysis of participants’ statements led to the identification of three main thematic areas relevant to the subject of this study. These categories naturally emerged from the data as clusters of frequently recurring themes and experiences that influence not only cultural perceptions but also nutritional decisions, event choice, and the recovery process after exertion. The three areas are (1) food as an element of cultural exploration, (2) local cuisine as a motivator or barrier to race selection from a nutritional perspective, (3) food as a symbolic reward and structured recovery practice supporting nutritional and psychological processes. This classification reflects distinct yet interconnected dimensions of runners’ experiences, enabling a comprehensive understanding of the significance of culinary practices and nutrition in sports tourism. By organizing the data in this way, the analysis addresses practical and emotional aspects, facilitating the clear interpretation and communication of the findings. Each of these topics is discussed in the following section, illustrated with quotes from the study participants.

### 3.1. Food as an Element of Cultural Exploration

For many participants, food constituted an important part of the broader experience associated with participating in sports competitions abroad. Their statements indicate that consuming local dishes was not only a practical aspect related to nutrition after physical exertion but also a cognitive and cultural act. In this view, food was treated as a form of engagement with the locality—a way to encounter otherness, become closer to the community, and even understand a place.

The respondents from the United Kingdom often saw local cuisine as an integral part of exploring the country, sometimes even as a substitute for other forms of cultural tourism. One participant emphasized “I love trying local flavors after the race. I don’t plan in advance what I’ll eat, but I always look for something typical of the place.” In this case, food is treated as a spontaneous adventure where taste becomes a gateway to broader discovery. Another participant added “Food tells me a lot about people—in Poland, I ate things I’d never seen in England, and it was great.” This approach points to an anthropological dimension of nutrition—food serves as a cultural medium for runners, carrying information about customs, history, and the mentality of the country’s inhabitants. A third United Kingdom respondent noted “I don’t always have time to visit museums, but I always find a moment to try local food. It’s the easiest way to get to know a place.” In this perspective, cuisine not only complements but even replaces other forms of cultural exploration, serving as a simple, accessible, and emotionally engaging means to explore space and community.

The participants from Ukraine also referred to food as part of cultural immersion, but their approach was more often characterized by a sense of soft cultural proximity. Polish cuisine was familiar to them, which eased their reception and reduced the risk of cultural discomfort, while still not excluding cognitive curiosity. One female participant stated “For me, food is also an opportunity to get to know the country. Polish cuisine is similar to ours, so I felt at home, but I happily tried dishes I didn’t know—like sour rye soup.” Such statements show that culinary exploration can also occur through subtle differences—seeking “familiarity, but different.” Another female respondent from Ukraine added “Running is just one element. In Poland, I feel quite at home, but I always discover something new.” This approach reveals an interesting dynamic: the simultaneous experience of familiarity and novelty, in which the culinary culture of the host country functions as a culturally refracted representation of known elements, offering athletes both continuity and reinterpretation within a new gastronomic context.

It is worth noting that the participants from Germany expressed a somewhat different motivation—more conscious, structured, and linked to intentionally extending their stay in the host country. One respondent stated directly “I have very strict personal dietary rules, during competitions I always stay an extra day or two to explore and try the local cuisine—this is part of the adventure, not just the competition.” This approach combines athletic discipline with openness to culinary experiences—where exploration of local cuisine is not spontaneous but planned. Another German participant even emphasized the aesthetic and cognitive dimension of nutrition: “I’m a fan of regional cuisines, and races are an opportunity for culinary exploration. I don’t treat food just functionally—I want it to taste good and to say something about the place I’m in.” Such statements align with broader trends of the “foodie traveler,” who treats food as a cultural text to be interpreted, not merely as a source of energy.

A common denominator for all participants—regardless of nationality—is that food functions as an interface between physical activity and the socio-cultural context of the host environment. They do not perceive their participation in competitions solely as a functional or technical trip; it is an opportunity for a broader experience of the country—with cuisine becoming one of the main channels of this experience. Interestingly, the differences between nationality groups mainly manifested in the way they experienced this exploration: the British preferred spontaneity and surprise, the Ukrainians valued cultural closeness and comfort, while the Germans were characterized by a planned, more reflective approach to culinary discovery.

In the context of sports tourism, these findings confirm that food can serve as a cultural interface, enabling quick contact with a locality without the need for deep immersion in language or traditions. In this sense, cuisine acts as a conduit for engaging with the local environment—opening access to the experience of a place in an accessible and satisfying way, especially for physically active people who have limited time and resources during a sports trip.

### 3.2. Local Cuisine as a Motivator or Barrier in Choosing a Race from a Nutrition Perspective

Although the primary criteria for selecting international running events remain sport-related factors—such as the course, climate, infrastructure, or the event’s reputation—the study results show that local cuisine can serve both as an additional motivation and as a limiting factor in the decision to participate. The participants’ statements indicate that food-related aspects are not entirely marginal—they can positively or negatively influence the overall attitude toward a destination, and in some cases even determine whether to cancel the trip altogether.

Among the German respondents, several comments highlighted the clear impact of cuisine on planning participation in foreign races. One respondent noted “When choosing international races, I somewhat pay attention to the food. For example, I’m happy to go to Italy because I love pizza and I know I’ll eat well after the race. Poland wasn’t on my list of ‘culinary’ destinations, but I have a positive opinion about it.” This statement shows that cuisine can act as an incentive, enhancing the appeal of certain locations—especially those already perceived in the tourist imagination as gastronomically attractive destinations. In this context, Poland was described as “neutral”—neither off-putting nor exceeding high expectations. A similar tone is echoed in another German runner’s remark: “I once ran a race in New York and the food exhausted me. Since then, I pay attention to whether I’ll have access to familiar dishes after the race. Poland was neutral—not impressive, but not off-putting either.” Here, it is clear that negative culinary experiences in the past can lead to a more cautious approach toward future trips. Thus, cuisine can act as a safety filter—a marker of comfort and predictability.

On the other hand, the participants from the United Kingdom emphasized the role of food as a factor that could support (or weaken) their decision to participate in a race to varying degrees. One participant said “It’s funny, but believe me, there are races I avoid because I know it’s hard to find decent food there. Poland wasn’t an obvious choice for me in terms of cuisine—I was more guided by the route and the atmosphere. Before the trip, I read that the food there is questionable and of low quality.” Although cuisine was not the main criterion here, its negative potential could influence the decision to skip certain locations. Another respondent added “Some of my friends choose races in cities where there’s ‘something to eat.’ It’s a bit funny but also logical—if you’re going anyway, you want to eat well after the effort.” This observation suggests the existence of informal running food maps—community preferences where food becomes part of the attraction package, not just a logistical backdrop. Another United Kingdom participant expressed disappointment with a specific city in Poland: “In big cities like Poznan, I expected more culinary variety. It’s a shame that the options were limited, at least for my tastes and preferences. For me, that’s one of the downsides of that location.” Such statements point to growing expectations regarding the culinary infrastructure of sporting events, especially in larger and popular tourist cities.

The statements from the Ukrainian participants reveal a somewhat different, more pragmatic attitude toward cuisine as part of race planning. One female respondent noted “I don’t choose races based on food, but I admit that if I know the cuisine will be familiar, I feel less stressed. Poland and Ukraine are similar, so it was easier for me.” For her, culinary predictability, rather than flavor appeal, was important as it lowered the level of uncertainty during the trip. Another participant added “I don’t think about food when planning a race, but when I thought about trying local delicacies after the run, I thought that was nice.” This quote suggests that the culinary aspect may gain significance secondarily—as a pleasant complement enriching the overall experience, though not a decisive factor. Among the Ukrainian participants, there were neither strong expectations nor critical opinions—local cuisine appeared more as a safe, familiar background rather than an attractive feature in its own right.

In the analyzed statements, three complementary patterns of approaching cuisine as a factor influencing race decisions emerge. The first is culinary pragmatism—participants prioritize securing familiar, comfortable food after the race (e.g., Germans). The second is culinary instrumentalization, where cuisine becomes a bonus or even a condition for participation, especially in locations with limited access to “decent food” (e.g., the British). The third is culinary neutrality with the potential for surprise—as seen among Ukrainians, for whom food is not a decisive factor but can have a positive effect during the event.

The results therefore show that local cuisine plays an ambivalent role: it can encourage, deter, or remain in the background, but it is rarely completely overlooked in the evaluation of a sporting event. Especially in countries like Poland, which are not automatically associated with globally recognized cuisine, culinary experiences can provide added value—provided that participants discover them on-site. It is worth noting that a destination’s culinary reputation affects not only expectations but also the willingness to explore them and, in this sense, it can represent a potential area of action for race organizers, local gastronomy, or regional promotional institutions.

### 3.3. Food as a Symbolic Reward and Structured Recovery Practice Supporting Nutritional and Psychological Processes

Many participants indicated that eating after a race serves a ritual function—it is not only an element of physical recovery but also a psychological closure of the effort. The participants’ statements suggest that the post-race meal can be both a symbol of reward and a way to regain balance, often in an emotional, almost homely dimension. In this context, food gains a personal and intimate meaning—it becomes not only nourishing but also significant.

Some respondents explicitly mentioned the need for a nourishing and warm meal after an intense run. A German participant emphasized “After an intense run, I need something warm and nourishing. It’s not just about calories but about psychological comfort. Local cuisine can really help here—if you find the right place.” This statement highlights the need for a sense of safety and soothing that a well-prepared, familiar, or emotionally comforting meal can bring. A similar meaning appears in the account of a runner from Ukraine: “I always have my routine—good hydration and a solid meal. In Poznan, I ate a homemade dinner, pork schnitzel with potatoes, at a small restaurant, and I felt like I was at my grandmother’s. For me, that’s also part of recovery—not just physical.” In this case, recovery takes on a sentimental dimension—the meal not only nourishes but also evokes emotional associations and private history.

Eating after a race often also serves as a form of reward—a permission to enjoy a moment of pleasure and a break from everyday dietary rules. One respondent from the United Kingdom said “I always eat something nice after a race that I normally wouldn’t allow myself. Recently, it was sweet pancakes. They were amazing! The taste of childhood.” Similarly, a participant from Ukraine shared “Before the race, I take care of my form, but afterwards I reward myself with different things. Sometimes it’s fast food, other times something local, usually fatty and unhealthy, but once in a while, it’s okay.” Both of these statements clearly point to the existence of a compensatory pattern—physical effort justifies culinary indulgence, often outside the boundaries of healthy eating. In this context, food is not just a biological necessity—it gains symbolic meaning, becoming a ritual of comfort, reward, and return to balance.

Among the participants, there were also more varied and personal statements, showing how individual recovery strategies can be. One German participant noted “A good meal is a symbol of closure for me—I recently had soup from a local restaurant and I was very satisfied.” Meanwhile, a respondent from the United Kingdom remarked “Sometimes the atmosphere after the race combined with good food gives more joy than the medal itself. In Poznan, it wasn’t perfect, but I found a nice little café with coffee and sweet treats, and that will stay with me.” In these statements, the meal appears as an element closing the sports ritual—especially when combined with the place, atmosphere, and company.

It is worth noting, however, that not all opinions were unequivocally positive. One participant from the United Kingdom expressed a critical view of Polish cuisine: “Polish food is quite fatty, and when I ran in Poznan, I didn’t really take advantage of it; I preferred a traditional chicken salad, which I eat regularly.” Similarly, a participant from Ukraine noted that although she enjoys spending time at the table, she does not attach much importance to Polish cuisine, which is too familiar to her: “I like sitting with someone, chatting, eating something tasty. But Polish cuisine is very similar to Ukrainian, so I don’t put much weight on it.” These statements show that personal culinary preferences and habits can influence whether local food is even considered as part of recovery or pleasure. For some participants, consistency and predictability are more important than exploring new flavors.

Eating after a race serves multidimensional functions: it can be a reward, a form of regeneration, a symbol of finishing the effort, as well as an emotional ritual of returning to comfort. Importantly, it is not only the type of food that determines its significance but also the context, atmosphere, and personal associations. Local cuisine can enrich this experience—if it suits the participant’s taste and is appropriately integrated into their rituals.

## 4. Discussion

The results of the conducted research indicate that the nutritional aspect in sports tourism should be considered not only in functional and physiological terms but also as a cultural, emotional, and identity-related phenomenon. Nutrition proves to be a multi-layered component of sports mobility, co-creating the experience of participating in a race abroad. This expanded understanding aligns with broader perspectives in tourism studies, where food is increasingly recognized as more than sustenance. Studies on the culinary dimension of tourism suggest that meals play a central role in how people experience and interpret a destination, as demonstrated by, among others, Jiménez-Beltrán et al. [47], Sims [48], and Stone and colleagues [49].

The collected statements confirm these findings, showing that for many participants, food constitutes an integral cognitive and symbolic element. Some respondents consciously treat meals as a medium of exploration—speaking about food as a way to get to know a country or as a tool for cultural contact. This approach corresponds with the concept of foodscapes [50,51,52], where the culinary landscape is not just a backdrop but an active component of the tourist experience. However, unlike studies focused on culinary tourism, where food often serves as the central motivation for travel [53,54], in the case of sports tourism, it plays a more peripheral, complementary—yet still significant—role.

Interestingly, this study shows that the experience of nutrition takes different forms depending on the participants’ country of origin. The respondents from Ukraine reported a greater cultural affinity with Polish cuisine, which reduced the stress related to eating after the race. This phenomenon aligns with the concept of cultural culinary comfort [55,56], according to which familiarity with tastes and food textures can positively influence the emotional reception of a destination. Meanwhile, the participants from the United Kingdom and Germany more often treated food as a potential source of risk or disappointment, confirming earlier observations that a lack of culinary predictability can act as a tourism barrier [57]. Interestingly, there were also reports of avoiding specific events due to the poor reputation of local cuisine—a rare situation in sports tourism research but known from the literature on food neophobia in travel [58,59,60].

The emotional aspect of eating was described as a “reward,” a “closure,” or a “taste of childhood.” In this way, nutrition transcended functional logic and entered the domain of the symbolic self-regulation of emotions and the moral justification of pleasure. Such mechanisms are discussed, among others, in counter-movements within fitness culture, where planned dietary deviations permit a temporary relaxation of strict regimen without eliciting feelings of guilt [61,62]. The respondents themselves admitted that after the race individuals often grant themselves dietary permissiveness—which points to the presence of a moral economy of eating [63].

In this context, it is worth noting that while the literature on sports nutrition mainly emphasizes the regenerative, metabolic, and technical aspects [64,65], the results of this study indicate a much broader range of meanings attributed to food after a race. Nutrition becomes part of a post-exertion ritual where the goal is not only energy restoration but also the psychological closure of the race experience [66]. The participants seek food characterized by traditional preparation, comforting qualities, and familiar flavors, suggesting a need for emotional grounding after intense effort. Thus, the nutrition aspect should be understood holistically—intertwined with emotions, autobiographical memory, and the symbolic order of exertion.

Moreover, there is a clear differentiation in nutritional strategies—some participants prefer flexibility and discovering new tastes, while others show attachment to routine and low-risk products. This phenomenon confirms findings from previous research on nutritional identities, which indicate that food choices are rooted not only in knowledge about food but also in lifestyles, class affiliation, and prior consumer practices [67,68]. This study clearly shows that participants are not a homogeneous group—they differ in their openness to local cuisine, level of culinary curiosity, and readiness to adapt.

It is also worth noting that although food played an important role in perceiving the value of the destination, it was not a decisive factor for everyone. Some respondents indicated that the choice of race was primarily based on the route and atmosphere, and that food-related considerations either emerged later or were only consciously recognized during the interview process. Thus, nutrition appears as a secondary factor but one that influences the overall evaluation of the event and the potential desire to return—which is also confirmed by studies on the role of food in building recreational tourism experiences [69,70]. Food does not have to be the main reason for the trip, but its quality can significantly enhance or weaken the overall impression of the journey.

## 5. Study Limitations and Practical Implications

While this study provides valuable insights into the nutritional experiences of runners participating in an international race, several contextual and methodological considerations should be noted. As is characteristic of qualitative research, the findings are context-bound and not intended for statistical generalization. This study relied on participants’ self-reported accounts, which may reflect subjective interpretations shaped by memory, perception, and emotional states. However, such subjectivity is an inherent strength of qualitative inquiry, as it enables access to rich, situated meaning making that would be difficult to capture through quantitative tools alone. The sample, though purposively diverse, included eight men and four women, which resulted in a gender imbalance that should be noted as a contextual limitation. Additionally, the participants were drawn from two high-income countries (Germany and the United Kingdom) and one low-income country (Ukraine). This socioeconomic contrast may have influenced individual experiences and perceptions of food, though the study did not aim to compare responses across economic lines. This limited cultural and structural spectrum may affect the transferability of the findings to other contexts, especially those involving runners from regions with distinct culinary practices, such as Asia, Latin America, or Africa. Nevertheless, transferability rather than generalizability is the key criterion here, and detailed descriptions of participant backgrounds and context allow readers to judge the study’s relevance to other settings. Furthermore, the event-specific nature of the research—focusing solely on the Poznan Half Marathon—introduces contextual embeddedness, which may limit the applicability to different races or locations. However, this focused scope also enables in-depth engagement with a specific phenomenon in a real-life setting, consistent with qualitative principles of contextual richness. No quantitative data were collected, but the study was designed to prioritize depth over breadth. Additionally, while factors such as weather, health conditions, or training loads were not explicitly analyzed, they may have implicitly shaped participant accounts. Future research incorporating these factors would help provide a more comprehensive picture of nutrition in the context of international sports tourism.

The findings of this study offer valuable practical insights for race organizers, local food providers, and tourism promoters. Recognizing that nutrition plays a multifaceted role—not only in physical recovery but also in emotional well-being and cultural experience—can help organizers design more holistic offerings that enhance participant satisfaction. Providing diverse, high-quality food options that cater both to familiar tastes and local specialties may encourage runners to engage more deeply with the event and its location, potentially increasing their likelihood of return participation. Moreover, understanding participants’ varying degrees of openness to local cuisine can guide tailored communication and menu planning, balancing comfort foods with opportunities for culinary exploration. Collaboration with local restaurants and food vendors can also serve to promote regional gastronomy as an added value of the sporting event, contributing to the local economy and enriching the overall experience. Ultimately, integrating thoughtful nutritional strategies into race planning can transform post-race meals from mere refueling into meaning.

## 6. Conclusions

The findings of this study underscore the complex and multidimensional function of nutrition within the context of international running events, extending beyond mere physiological requirements. Nutrition is revealed not only as a critical factor in physical recovery but also as a significant emotional and symbolic element of the athlete’s experience, influencing perceptions of reward, comfort, and cultural affiliation. The role of local cuisine in enhancing the overall appeal of the event is evident; however, its effects are modulated by individual differences in preferences, cultural backgrounds, and previous exposure to regional foods. These findings indicate that event organizers and stakeholders in sports tourism should adopt a more nuanced and culturally responsive approach to nutritional provision, aiming to address both the physical and psychosocial needs of participants. Ultimately, framing nutrition as an integrative and culturally situated aspect of sports tourism can facilitate richer participant experiences, promote repeat engagement, and enhance the host destination’s reputation.

## Data Availability

The original contributions presented in the study are included in the article; further inquiries can be directed to the corresponding author.
